# High expression of DOCK2 indicates good prognosis in acute myeloid leukemia

**DOI:** 10.7150/jca.33244

**Published:** 2019-10-15

**Authors:** Ning Hu, Yifan Pang, Hongmian Zhao, Chaozeng Si, Hui Ding, Li Chen, Chao Wang, Tong Qin, Qianyu Li, Yu Han, Yifeng Dai, Yijie Zhang, Jinlong Shi, Depei Wu, Xinyou Zhang, Zhiheng Cheng, Lin Fu

**Affiliations:** 1 Department of Hematology, Huaihe Hospital of Henan University, Kaifeng, 475000, China; 2 Department of Medicine, William Beaumont Hospital, Royal Oak, MI 48073, USA; 3 Department of Operations and Information Management, China-Japan Friendship Hospital, Beijing, 100029, China; 4 Department of Pathology and Medical Biology, University Medical Center Groningen, University of Groningen, Groningen, Netherlands; 5 Department of Respiratory, Huaihe Hospital of Henan University, Kaifeng, 475000, China; 6 Department of Biomedical Engineering, Chinese PLA General Hospital, Beijing, 100853, China; 7 Department of Hematology, The First Affiliated Hospital of Soochow University, Suzhou 215006, China; 8 Department of Hematology, The Second Clinical Medical College (Shenzhen People's Hospital), Jinan University, Shenzhen 518020, China; 9 Department of Hematology, The Second Affiliated Hospital of Guangzhou Medical University, Guangzhou, 510260, China; 10 Translational Medicine Center, The Second Affiliated Hospital of Guangzhou Medical University, Guangzhou, 510260, China

**Keywords:** acute myeloid leukemia, DOCK2, allogeneic hematopoietic stem cell transplantation, chemotherapy, prognosis

## Abstract

DOCK family proteins are evolutionarily conserved guanine nucleotide exchange factors for Rho GTPase with different cellular functions. It has been demonstrated that DOCK1 had adverse prognostic effect in acute myeloid leukemia (AML). We first analyzed data of 85 AML patients who were treated with chemotherapy and had available DOCK1 to DOCK11 expression information and found that DOCK1 and DOCK2 had prognostic significance in AML. In view of the known prognosis of DOCK1 in AML, we then explored the prognostic role of DOCK2. One hundred fifty-six AML patients with DOCK2 expression data were extracted from The Cancer Genome Atlas (TCGA) database and enrolled in this study. Patients were divided based on treatment modality into the chemotherapy group and the allogeneic hematopoietic stem cell transplant (allo-HSCT) group. Each group was divided into two groups by the median expression levels of DOCK2. In the chemotherapy group, high DOCK2 expression was associated with longer event-free survival (EFS, *P*=0.001) and overall survival (OS, *P*=0.007). In the allo-HSCT group, EFS and OS were not significantly different between high and low DOCK2 expression groups. Multivariate analysis showed that high DOCK2 expression was an independent favorable prognostic factor for both EFS and OS in all patients (all *P*<0.05). In conclusion, our results indicated that high DOCK2 expression, in contrast to DOCK1, conferred good prognosis in AML.

## Introduction

Genetic abnormality is not only the pathogenic basis of acute myeloid leukemia (AML) 1 but also has important prognostic implications. For example, *NPM1* mutations and double *CEBPA* mutations are associated with favorable prognosis in cytogenetically normal AML (CN-AML) 2, 3, while *DNMT3A* and *WT1* mutations are adverse prognostic factors 4, 5.

The dedicators of cytokinesis (DOCK) family, including DOCK1 to DOCK11 proteins, are evolutionarily conserved guanine nucleotide exchange factors (GEF) for the Rho GTPases Rac. It is involved in various pathologies including cancers, immune disorders, and central nervous system diseases 6. For instance, high DOCK1 expression is an unfavorable prognostic marker in breast cancer and ovarian cancer 7,8, and it induces migration and invasion of malignant cells in lung and brain cancer 9, 10. Fukui Y et al found that DOCK2 is only expressed in hematopoietic tissues 11. In addition, DOCK2 is also associated with the development of various cancers 12.

Previous study has shown that high DOCK1 expression conferred poor prognosis in AML 13, but the prognosis value of the other DOCK family members in AML is unclear. We screened all the DOCK family members and found that DOCK2 also had independent prognostic significance in AML.

## Materials and Methods

### Patients

From The Cancer Genome Atlas (TCGA) database (https://cancergenome.nih.gov/), 156 AML patients with DOCK2 expression data were enrolled in this study 14. All patients were between ages 18 and 88, registered between November 2001 and March 2010, were selected from a set of more than 400 samples to reflect a realworld distribution of subtypes. Eighty-five patients were treated with chemotherapy alone, and other 71 received allo-HSCT. Patients treated with chemotherapy alone were defined as the chemotherapy group; patients who underwent allo-HSCT were defined as the allo-HSCT group. Then, each group was divided into two subgroups by the respective median DOCK2 expression levels. All clinical and molecular information including DOCK2 expression levels were publicly accessible from the TCGA website. All patients provided written informed consent and the research was approved by the Human Research Ethics Committee of Washington University. Primary endpoints were event-free survival (EFS) and overall survival (OS). EFS was defined as the time from diagnosis to removal from the study due to the absence of complete remission, relapse or death or was censored at the last follow-up. OS was defined as the time from diagnosis to death or was censored at the last follow-up.

### Statistical Analysis

The clinical and molecular characteristics of patients were summarized using descriptive statistics. The Mann-Whitney *U* test and the chi-square test were used to compare continuous and categorical data, respectively. EFS and OS were estimated with the Kaplan-Meier method and compared using the log-rank test. Cox proportional hazard model was constructed for EFS and OS to identify possible prognostic factors among the clinical and molecular variables. All statistical analyses were performed by SPSS software 20.0 and GraphPad Prism software 5.0. For all statistical analyses, *P-*values were two-sided and *P*<0.05 was considered significant.

## Results

### Comparison of EFS and OS between different expression levels of DOCK1*-*11

To assess the prognostic significance of DOCK family in AML, EFS and OS patients with high and low expression groups of each DOCK family proteins were compared (Table 1). The results showed that DOCK1 was an adverse prognostic factor and DOCK2 was a favorable prognostic factor in AML. However, other DOCK members had no effect on EFS and OS.

### Association between DOCK2 expression and patient's characteristics

Comparison of clinical and molecular characteristics between different expression subgroups within chemotherapy and allo-HSCT groups were summarized in Table 2. In the chemotherapy group, high DOCK2 expression group had more good-risk patients (*P*=0.014), fewer poor-risk patients (*P*=0.002) and less *TP53*mutations (*P*=0.049) than low expression group. Six patients among the low expression group harbored *CBFβ-MYH11*, which was not found in the high expression group (*P*=0.012). No significant difference was found in age, sex distribution, peripheral white blood cell (WBC) count and bone marrow blast (BM) percentage at diagnosis, French-American-British (FAB) classification, frequency of other recurrent genetic mutations (*FLT3-ITD*, *NPM1*, *CEBPA*, *IDH1*/*IDH2*, *RUNX1*, *MLL-PTD*, *NRAS*/*KRAS*, *TET2*, *WT1* and* TP53*), or relapse rate between the high and low expression subgroups.

In the allo-HSCT group, high DOCK2 expression group had fewer poor-risk patients (*P*=0.007), fewer normal karyotype patients (*P*=0.005), more *CEBPA* (*P*=0.033) and *DNMT3A* mutations (*P*=0.044) than the low expression group. No significant difference was found in age, sex distribution, BM blasts, FAB classification, frequent AML mutations (*FLT3-ITD*, *NPM1*, *IDH1*/*IDH2*, *RUNX1*, *MLL-PTD*, *NRAS*/*KRAS*,* TET2*, *WT1* and* TP53*), or relapse rate between two subgroups.

### Prognostic value of DOCK2 in AML

**Figure 1:**
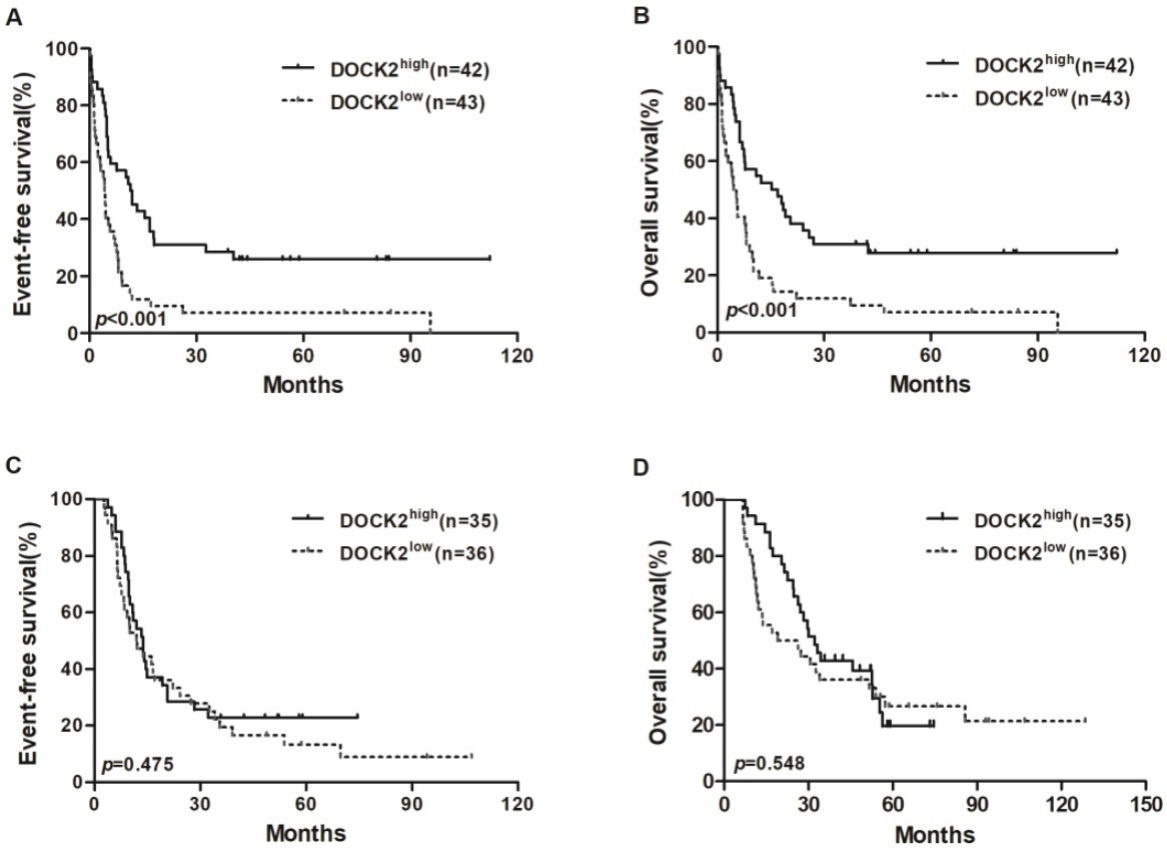
** Kaplan-Meier curves of EFS and OS in the chemotherapy and allo-HSCT groups.** (A, B) In the chemotherapy group, high DOCK2 expressers had longer EFS and OS than low expressers. (C, D) EFS and OS were not significantly different between high and low DOCK2 expression subgroups in the allo-HSCT group.

In the chemotherapy group, high DOCK2 expressers had longer EFS and OS (all *P*<0.001; Figure 1A and 1B) than low expressers, but survival was not significantly different between DOCK2 high and low expression subgroups in the allo-HSCT group (Figure 1C and 1D).

We chose DOCK2 expression levels (low vs. high), therapy method (chemotherapy vs. allo-HSCT), age (<60 vs. ≥60 years), WBC counts (<20×10^9^/L vs. ≥20×10^9^/L), *FLT3-ITD* (positive vs. negative) and common AML mutations (*NPM1*, *DNMT3A*, *IDH1*/*IDH2*, *RUNX1*, *WT1*, *CEBPA* and *TP53*, mutated vs.wild) to construct multivariate analysis of EFS and OS.

In the chemotherapy group, multivariate analysis showed that age ≥60 years and *TP53* mutations were independent risk factors for EFS and OS (all *P*<0.05), and high DOCK2 expression was an independent favorable factor for EFS and OS (all *P*<0.05, Table 3). In the allo-HSCT group, multivariate analysis showed that *FLT3-ITD* was an independent risk factor for EFS and OS (all *P*<0.05). WBC counts ≥20×10^9^/L and *WT1* mutations were independent risk factors for EFS. Mutations in *RUNX1* and *TP53* were independent risk factors for OS (all* P*<0.05, Table 4).

In all patients, multivariate analysis showed that high DOCK2 expression and allo-HSCT were independent favorable factors for EFS and OS (all *P*<0.05). Age ≥60 years, WBC counts ≥20×10^9^/L and mutations in *DNMT3A, RUNX1* and *TP53* were independent risk factors for EFS and OS (all *P*<0.05, Table 5).

### Correlation analysis of DOCK2 expression and genome-wide microRNA and gene expression profile

**Figure 2:**
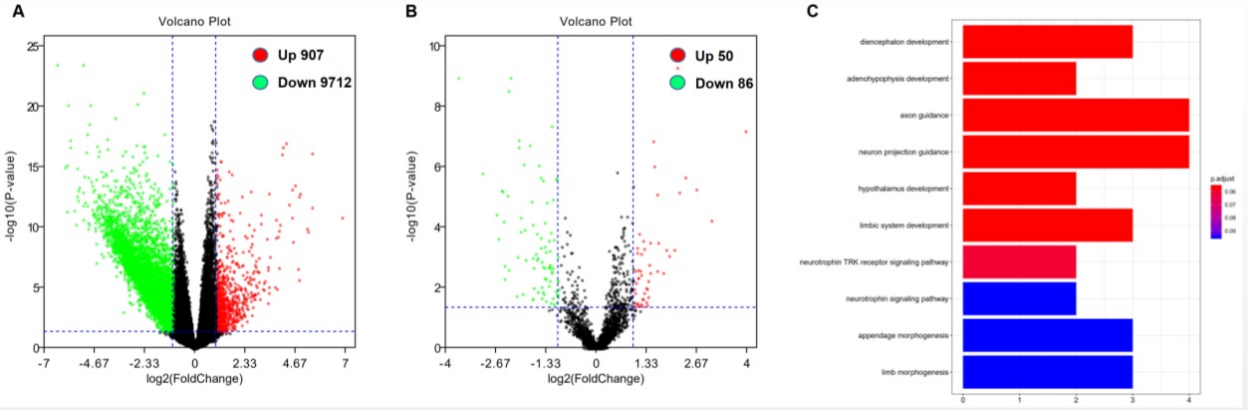
** Genome-wide gene/microRNA expression profiles and cell signaling pathways associated with DOCK2 expression.** (A) Volcano plot of differential gene expression. Up-regulated and down-regulated genes were labeled with red and green dots, respectively. (B) Volcano plot of differential microRNA expression. Up-regulated and down-regulated microRNAs were labeled with red and green dots, respectively. (C) Gene ontology (GO) enrichment analysis of genes related to DOCK2 expression.

In order to further evaluate the role of DOCK2 in AML, we obtained DOCK2-associated gene expression profiles and mircroRNA from TCGA database through high-throughput sequencing. There were 907 genes were positively associated with DOCK2 expression, and 9712 genes were negatively associated with DOCK2 expression (*P*<0.05, fold change=1.5, Figure. 2A). Then, we identified 50 up-regulated and 86 down-regulated microRNAs that were significantly correlated with DOCK2 expression (*P*<0.05, fold change=1.5, Figure. 2B). Furthermore, gene ontology (GO) enrichment analysis suggested that the genes related to DOCK2 expression were mainly concentrated in "diencephalon development", "adenohypophysis development", "axon guidance", "neuron projection guidance", "hypothalamus development", "limbic system development", "neurotrophin TRK receptor signaling pathway", "neurotrophin signaling pathway", "appendage morphogenesis", and "limb morphogenesis" pathways (Figure. 2C).

## Discussion

Our study showed that high DOCK2 expression was an independent favorable factor in AML patients who underwent chemotherapy alone, but not in patients who also underwent allo-HSCT. Consistent with previous studies, we also found that high DOCK1 expression was an adverse factor in AML 13.

Previous researches have demonstrated that *TP53* mutation and older age were negative prognostic factors in AML 15,16, while *CBFβ-MYH11* was associated with favorable prognosis in AML 17. Our study found that in high DOCK2 expression patients, there were more good-risk patients, more *CBFβ-MYH11*, and fewer *TP53* mutations, suggesting that high expression of DOCK2 was more likely to co-exist with *CBFβ-MYH11* rather than *TP53* mutations. In the chemotherapy group, the survival analysis indicated that high DOCK2 expression was a favorable factor for EFS and OS, but it not in the allo-HSCT group, suggesting that the unfavorable effect of low DOCK2 expression might be overcome by allo-HSCT.

DOCK2 has been shown to be a specific Rac activator in mature lymphocytes 18. It is involved in neutrophil chemotaxis 19 and NK cells differentiation 20. Previous study found that DOCK2 plays a key role in the regulation of cell proliferation in diffuse large B cell lymphoma and follicular lymphoma via the ERK signaling pathway 21. Nishihara H et al found that DOCK2 is associated with CrkL and regulates Rac1 in human leukemia cell lines 22. Another study revealed that DOCK2 regulates CXCR4 signaling in immature hematopoietic cells 23. In the present study, DOCK2 was associated with "neurotrophin TRK receptor signaling pathway", "neurotrophin signaling pathway", "appendage morphogenesis". We speculate that DOCK2 may play a prognostic role in leukemia by interacting with genes in these functional pathways.

A previous study suggested that knocking down DOCK2 could sensitize *FLT3-ITD* leukemic cells to cytarabine treatment through the inhibition of Rac1 pathway 24, whereas in this study, we observed a favorable prognostic impact of high DOCK2 expression in AML patients. This discrepancy might be related to the different research objects of the two studies*,* since we did not specifically study AML patients with *FLT3-ITD*.

DOCK2 may play different roles in the lymphoid and myeloid system 21. This is similar to LEF1. High LEF1 expression has been reported as a favorable prognostic factor in CN-AML 25, but it is also an adverse prognostic factor in adult B-precursor acute lymphoblastic leukemia 26. Low expression of DOCK2 is associated with poorer prognosis in colorectal cancer 27. However, the expression level of DOCK2 is positively correlated with the proliferation rate of CXCL13-induced prostate cancer cells 28. We theorized that DOCK2 had tissue-specific effects in different malignancies.

In summary, two of the 11 members of the DOCK family have prognostic significance in AML. DOCK1 has adverse prognostic effect and DOCK2 the opposite. This finding may further deepen the risk stratification system of AML.

## Figures and Tables

**Table 1:** Comparison of EFS and OS between different expression levels of Dock1-11 based on chemotherapy.

Variables	EFS			OS	
	χ^2^	*P*-value		χ^2^	*P*-value
Dock1 (high vs. low)	14.908	0.000		14.343	0.000
Dock2(high vs. low)	13.331	0.000		11.748	0.001
Dock3 (high vs. low)	0.030	0.863		0.000	0.999
Dock4(high vs. low)	1.598	0.206		1.658	0.198
Dock5(high vs. low)	0.153	0.695		0.021	0.884
Dock6(high vs. low)	0.930	0.335		0.312	0.576
Dock7 (high vs. low)	0.552	0.457		1.261	0.262
Dock8(high vs. low)	0.288	0.591		0.419	0.518
Dock9(high vs. low)	0.170	0.680		0.497	0.481
Dock10(high vs. low)	0.011	0.916		0.009	0.923
Dock11(high vs. low)	0.054	0.817		0.002	0.968

**Abbreviations:** EFS, event-free survival; OS, overall survival.

**Table 2:** Clinical and molecular characteristics of patients according to DOCK2 levels

Characteristics	Chemotherapy group				Allo-HSCT group		
	High DOCK2 (n = 42)	Low DOCK2 (n = 43)	*P*		High DOCK2 (n = 35)	Low DOCK2 (n = 36)	*P*
Age/years, median (range)	66.5 (22-77)	66 (33-88)	0.324^*^		51 (22-69)	52.5 (18-72)	0.890^*^
Age group/n (%)			0.311^§^				0.205^§^
< 60 years	15 (35.7)	11 (25.6)			28 (80.0)	24 (66.7)	
≥ 60 years	27 (64.3)	32 (74.4)			7 (20.0)	12 (33.3)	
Gender/n (%)			0.591^§^				0.288^§^
Male	21 (50.0)	24 (55.8)			18 (51.4)	23 (63.9)	
Female	21 (50.0)	19 (44.2)			17 (48.6)	13 (36.1)	
WBC/×10^9^/L, median (range)	15.2(1.0-171.9)	12.3(0.7-297.4)	0.329^*^		30.9(1.2-223.8)	27.7(0.6-90.4)	0.200^*^
BM blast/%, median (range)	71 (30-97)	74 (32-99)	0.379^*^		71 (34-100)	70 (30-97)	0.809^*^
PB blast/%, median (range)	23 (0-91)	25 (0-98)	0.972^*^		48 (0-96)	53 (0-90)	0.801^*^
FAB subtypes/n(%)							
M0	4 (9.5)	3 (7.0)	0.713^§^		3 (8.6)	6 (16.7)	0.478^§^
M1	7 (16.7)	13 (30.2)	0.140^§^		14 (40.0)	9 (25.0)	0.177^§^
M2	12 (28.6)	9 (20.9)	0.414^§^		8 (22.9)	10 (27.8)	0.634^§^
M3	0 (0.0)	0 (0.0)			0 (0.0)	1 (2.8)	1.000^§^
M4	11 (26.2)	9 (20.9)	0.568^§^		8 (22.9)	5 (13.9)	0.329^§^
M5	7 (16.7)	6 (14.0)	0.728^§^		1 (2.9)	3 (8.3)	0.614^§^
M6	1 (2.4)	0 (0.0)	0.494^§^		0 (0.0)	1 (2.8)	1.000^§^
M7	0 (0.0)	2 (4.7)	0.494^§^		0 (0.0)	1 (1.4)	1.000^§^
Karyotype/n(%)							
Normal	21 (50.0)	19 (44.2)	0.591^§^		11 (29.7)	23 (62.2)	0.005^§^
Complex	3 (7.1)	9 (20.9)	0.117^§^		7 (18.9)	5 (13.5)	0.528^§^
inv(16)/CBFβ-MYH11	6 (14.3)	0 (0.0)	0.012^§^		5 (13.5)	0 (0.0)	0.054^§^
11q23/MLL	0 (0.0)	3 (7.0)	0.241^§^		2 (5.4)	1 (2.7)	1.000^§^
t(15;17)/PML-RARA	0 (0.0)	0 (0.0)			1 (2.7)	1 (2.7)	1.000^§^
t(9;22)/BCR-ABL1	0 (0.0)	1 (2.3)	1.000^§^		2 (5.4)	0 (0.0)	0.493^§^
t(8;21)/RUNX1-RUNX1T1	4 (9.5)	2 (4.7)	0.433^§^		0 (0.0)	1 (2.7)	1.000^§^
Others	8 (19.0)	9 (20.9)	0.828^§^		4 (10.8)	2 (5.4)	0.674^§^
Risk/n(%)							
Good	10 (23.8)	2 (4.7)	0.014^§^		5 (14.3)	2 (5.6)	0.260^§^
Intermediate	26 (61.9)	20 (46.5)	0.154^§^		23 (65.7)	17 (47.2)	0.116^§^
Poor	6 (14.3)	19 (44.2)	0.002^§^		6 (17.1)	17 (47.2)	0.007^§^
*FLT3-ITD*/n(%)			0.366^§^				0.730^§^
Presence	9 (21.4)	6 (14.0)			9 (25.7)	8 (22.2)	
Absence	33 (78.6)	37 (86.0)			29 (78.4)	28 (75.7)	
*NPM1*/n(%)			0.440^§^				0.246^§^
Mutation	15 (35.7)	12 (27.9)			11 (31.4)	7 (19.4)	
Wildtype	27 (64.3)	31 (72.1)			24 (68.6)	29 (80.6)	
*CEBPA*/n(%)			0.557^§^				0.033^§^
Single mutation	1 (2.4)	2 (4.7)			4 (11.4)	1 (2.8)	
Double mutation	0 (0.0)	0 (0.0)			3 (8.6)	0 (0.0)	
Wild type	41 (97.6)	41 (95.3)			28 (80.0)	35 (97.2)	
*DNMT3A*/n(%)			0.859^§^				0.044^§^
Mutation	11 (26.2)	12 (27.9)			12 (34.3)	5 (13.9)	
Wildtype	31 (73.8)	31 (72.1)			23 (65.7)	31 (86.1)	
*IDH1*/*IDH2*/n(%)			0.366^§^				0.730^§^
Mutation	9 (21.4)	6 (14.0)			9 (25.7)	8 (22.2)	
Wildtype	33 (78.6)	37 (86.0)			26 (74.3)	28 (77.8)	
*RUNX1*/n(%)			0.156^§^				0.260^§^
Mutation	6 (14.3)	2 (4.7)			2 (5.7)	6 (16.7)	
Wildtype	36 (85.7)	41 (95.3)			33 (94.3)	30 (83.3)	
*WT1*/n(%)			1.000^§^				0.478^§^
Mutation	1 (2.4)	1 (2.3)			5 (14.3)	3 (8.3)	
Wildtype	41 (97.6)	42 (97.7)			30 (85.7)	33 (91.7)	
*MLL-PTD*/n(%)			0.360^§^				0.614^§^
Presence	1 (2.4)	4 (9.3)			1 (2.9)	3 (8.3)	
Absence	41 (97.6)	39 (90.7)			34 (97.1)	33 (91.7)	
*NRAS/KRAS*/n(%)			0.505^§^				0.710^§^
Mutation	7 (16.7)	5 (11.6)			4 (11.4)	3 (8.3)	
Wildtype	35 (83.3)	38 (88.4)			31 (88.6)	33 (91.7)	
*TET2*/n(%)			0.778^§^				1.000^§^
Mutation	5 (11.9)	6 (14.0)			2 (5.7)	2 (5.6)	
Wildtype	37 (88.1)	37 (86.0)			33 (94.3)	34 (94.4)	
*TP53*/n(%)			0.049^§^				0.115^§^
Mutation	2 (4.8)	9 (20.9)			0 (0.0)	4 (11.1)	
Wildtype	40 (95.2)	34 (79.1)			35 (100.0)	32 (88.9)	
Relapse/n(%)			0.227^§^				0.614^§^
Yes	18 (42.9)	13 (30.2)			25 (71.4)	23 (63.9)	
No	24 (57.1)	30 (69.8)			10 (28.6)	13 (36.1)	

**Abbreviations:** WBC: white blood cell; BM: bone marrow; PB: peripheral blood; FAB: French American British. *denotes Mann-Whitney *U* test; §denotes chi-square test.

**Table 3:** Multivariate analyses for EFS and OS based on chemotherapy

Variables	EFS			OS	
	HR (95%CI)	*P*-value		HR (95%CI)	*P*-value
DOCK2 (high vs. low)	2.301 (1.381-3.835)	0.001		1.974 (1.201-3.245)	0.007
Age (< 60 v. ≥ 60 years)	2.909 (1.550-5.460)	0.001		2.582 (1.355-4.918)	0.004
WBC (<20 vs. ≥20×10^9^/L)	1.382 (0.777-2.457)	0.270		1.263 (0.719-2.220)	0.416
*NPM1,*mutated v wild type	0.653 (0.352-1.210)	0.175		0.813 (0.439-1.504)	0.509
*DNMT3A,* mutated v wild type	0.674 (0.375-1.211)	0.187		0.631 (0.357-1.117)	0.114
*FLT3-ITD,* presence v absence	0.801 (0.411-1.558)	0.512		0.974 (0.495-1.916)	0.939
*IDH1/IDH2,* mutated v wild typemutated v wild type	1.077 (0.555-2.089)	0.827		1.106 (0.560-2.185)	0.772
*RUNX1,* mutated v wild type	0.508 (0.218-1.185)	0.117		0.500 (0.214-1.168)	0.109
*WT1,* mutated v wild type	0.638 (0.134-3.041)	0.573		1.094 (0.134-8.929)	0.933
*CEBPA,* mutated v wild type	0.471 (0.139-1.596)	0.226		0.461 (0.136-1.567)	0.215
*TP53,* mutated v wild type	0.351 (0.154-0.801)	0.013		0.414 (0.184-0.933)	0.033

**Abbreviations:** EFS: Event-free survival; OS: Overall survival; WBC: white blood cell.

**Table 4:** Multivariate analyses for EFS and OS based on allo-HSCT.

Variables	EFS			OS	
	HR (95%CI)	*P*-value		HR (95%CI)	*P*-value
DOCK2 (high vs. low)	1.741 (0.921-3.294)	0.088		1.386 (0.705-2.725)	0.344
Age (< 60 v. ≥ 60 years)	0.869 (0.453-1.670)	0.674		1.174 (0.600-2.299)	0.639
WBC (<20 vs. ≥20×10^9^/L)	2.151 (1.127-4.105)	0.020		1.339 (0.687-2.612)	0.391
*NPM1,*mutated v wild type	1.878 (0.885-3.984)	0.101		1.476 (0.621-3.507)	0.378
*DNMT3A,* mutated v wild type	0.711 (0.344-1.468)	0.356		0.553 (0.258-1.183)	0.127
*FLT3-ITD,* presencevabsence	0.407 (0.201-0.826)	0.013		0.451 (0.206-0.990)	0.047
*IDH1/IDH2,* mutated v wild typemutated v wild type	0.800 (0.354-1.806)	0.591		1.058 (0.442-2.529)	0.900
*RUNX1,* mutated v wild type	0.822 (0.333-2.030)	0.671		0.386 (0.155-0.958)	0.040
*WT1,* mutated v wild type	0.361 (0.137-0.949)	0.039		0.607 (0.239-1.540)	0.293
*CEBPA,* mutated v wild type	1.358 (0.502-3.676)	0.547		1.106 (0.402-3.042)	0.846
*TP53,* mutated v wild type	0.371 (0.112-1.222)	0.103		0.155 (0.043-0.557)	0.004

**Abbreviations:** EFS: Event-free survival; OS: Overall survival; WBC: white blood cell.

**Table 5:** Multivariate analyses for EFS and OS based on chemotherapy and allo-HSCT.

Variables	EFS			OS	
	HR (95%CI)	*P*-value		HR (95%CI)	P-value
DOCK2 (high vs. low)	1.721 (1.175-2.518)	0.005		1.489 (1.012-2.191)	0.044
Chemotherapy v allo-HSCT	1.599 (1.097-2.330)	0.015		1.946 (1.301-2.910)	0.001
Age (< 60 vs. ≥ 60 years)	1.664 (1.107-2.500)	0.014		1.957 (1.270-3.016)	0.002
WBC (<20 vs. ≥20×10^9^/L)	1.649 (1.103-2.465)	0.015		1.331 (0.883-2.005)	0.172
*NPM1,* mutated v wild type	0.936 (0.593-1.477)	0.777		0.904 (0.559-1.461)	0.680
*DNMT3A,* mutated v wild type	0.578 (0.382-0.876)	0.010		0.536 (0.352-0.816)	0.004
*FLT3-ITD,* presence v absence	0.781 (0.488-1.252)	0.305		0.888 (0.533-1.478)	0.646
*IDH1/IDH2,* mutated v wild typemutated v wild type	1.135 (0.705-1.830)	0.602		1.229 (0.746-2.026)	0.419
*RUNX1,* mutated v wild type	0.535 (0.294-0.974)	0.041		0.413 (0.224-0.764)	0.005
*WT1,* mutated v wild type	0.612 (0.290-1.288)	0.196		0.762 (0.352-1.650)	0.491
*CEBPA,* mutated v wild type	0.580 (0.273-1.232)	0.156		0.671 (0.310-1.453)	0.312
*TP53,* mutated v wild type	0.310 (0.160-0.600)	0.001		0.269 (0.137-0.531)	0.000

**Abbreviations:** EFS: Event-free survival; OS: Overall survival; WBC: white blood cell.
